# Endoscopic Recurrence in Crohn’s Disease Patients With Long-Term Ileostomy

**DOI:** 10.1093/ibd/izaf153

**Published:** 2025-07-12

**Authors:** Lotte Oldenburg, Brecht Hens, Maxime Hollenberg, Geert D’Haens

**Affiliations:** Department of Gastroenterology and Hepatology, Amsterdam UMC, Location VUmc, Amsterdam Gastroenterology, Endocrinology & Metabolism, Amsterdam, The Netherlands; Department of Gastroenterology and Hepatology, Amsterdam UMC, Location VUmc, Amsterdam Gastroenterology, Endocrinology & Metabolism, Amsterdam, The Netherlands; Department of Gastroenterology and Hepatology, Vrije Universiteit Brussel (VUB), Universitair Ziekenhuis Brussel, Brussels, Belgium; Department of Gastroenterology and Hepatology, Amsterdam UMC, Location VUmc, Amsterdam Gastroenterology, Endocrinology & Metabolism, Amsterdam, The Netherlands; Department of Gastroenterology and Hepatology, Amsterdam UMC, Location VUmc, Amsterdam Gastroenterology, Endocrinology & Metabolism, Amsterdam, The Netherlands

**Keywords:** Crohn’s disease, ileitis, postoperative, ileostomy, natural history

## Abstract

**Background:**

A small but significant proportion of patients with Crohn’s disease (CD) will ultimately require a permanent ileostomy. So far, research has focused primarily on clinical and surgical recurrence rates in the ileum, leaving endoscopic recurrence largely unexplored. The aim of this study was to explore the endoscopic ileal recurrence rate in patients with a long-term ileostomy and to identify potential risk factors.

**Methods:**

We performed a retrospective study of adult CD patients with a long-term ileostomy (≥12 months) at a tertiary referral center.

**Results:**

Through an electronic health record database search, we were able to identify 150 patients. One hundred sixteen patients (77.3%) underwent at least one endoscopic examination of the ileum. Ileal recurrence was detected in 46/116 (39.7%). The 1, 3, and 5-year endoscopic recurrence rates were 11.2%, 27.3%, and 33.0%, respectively. Patients with earlier ileal involvement (hazard ratio [HR] 1.99, 95% confidence interval [CI] 1.09-3.62; *P* = .02) or previous biological therapy (HR 2.48, 95% CI 1.25-4.89; *P* = .01) were at higher risk. Fecal calprotectin in ileostomy effluent accurately predicted endoscopic inflammation at a cutoff of 170 mcg/g (sensitivity 77.8%, specificity 94.7%, accuracy 89.9%).

**Conclusions:**

In this retrospective study, 77.3% of ileostomy patients underwent endoscopic assessment during follow-up. Ileal recurrence was detected in 39.7% of patients who underwent endoscopic evaluation. Patients with ileal involvement or preoperative exposure to biologics had the highest risk of recurrence. These patients might benefit from endoscopic monitoring. Fecal calprotectin is a reliable noninvasive marker for detecting ileal inflammation.

**Key Messages**

**What is already known?**

A significant number of patients with Crohn’s disease (CD) will need permanent ileostomy during the course of their disease. Data on the postoperative natural history is limited to clinical or combined endpoints and little is known about the endoscopic recurrence rate in this specific patient group.

**What is new here?**

In this retrospective cohort study, endoscopic ileal recurrence was detected in one-third of patients within 5 years after ileostomy placement. Independent risk factors for developing ileitis were prior ileal Crohn’s disease and previous biological treatment, based on an endoscopically evaluated subgroup. Fecal calprotectin served as an excellent noninvasive biomarker for ileal inflammation.

**How can this study help patient care?**

Bio-experienced patients with prior ileal disease are at high risk of developing ileal recurrence after ileostomy placement and should undergo postoperative monitoring, including serial measurements of fecal calprotectin in ileostomy effluent every 6 months and endoscopy. Elevated calprotectin levels should prompt endoscopic evaluation to assess for ileostomy ileitis.

## Introduction

Crohn’s disease (CD) is a chronic, relapsing inflammatory disorder that can affect the entire gastrointestinal tract and has a significant impact on patients’ quality of life. Despite therapeutical advances, over a quarter of CD patients still require intestinal resections within 10 years of diagnosis.^1^ Surgery often proves noncurative, and up to one-third experience re-resection within the same timeframe.^1^

In patients with extensive and refractory CD, a subtotal colectomy or proctocolectomy with ileostomy is performed. Despite advanced medical therapies, the incidence of ostomy formation has not decreased since 2003, according to findings from a large Swedish cohort study.^2^ In most ileostomy cases, bowel continuity is restored within 6-12 months. Unfortunately, this is not always feasible. Patients with perianal CD, in particular, have a low success rate (only 17%) for successful bowel restoration. Consequently, up to 10% of patients may ultimately end up with a permanent ileostomy.^3,4^

Postoperative ileal recurrence in patients with an ileostomy remains a significant clinical challenge.^5^ A comprehensive systematic review and meta-analysis provided valuable insights into clinical recurrence, demonstrating a pooled recurrence rate of 28%.^2^ Although this study provides important data on clinical relapse, further research is needed to better understand the endoscopic disease course. Current data on endoscopic recurrence are limited, with existing studies often hampered by small sample sizes and use of combined endpoints.^2,6,7^

Current European guidelines advise to perform an ileocolonoscopy within 6-12 months after ileocecal surgery in patients with an ileocolonic anastomosis, and to start treatment in those with active disease.^8–10^ Besides, patients considered to be at high risk of recurrence can be started on early prophylactic therapy with a thiopurine or an anti-TNF agent.^9–11^ This lack of data in patients with a long-term ileostomy translates to a significant gap in postoperative monitoring and treatment strategies.

To address this critical knowledge gap and improve patient care, this study aims to investigate the natural history of endoscopic ileal recurrence following long-term ileostomy creation and identify potential risk factors.

## Materials and Methods

### Selection of Patients

This was a single-center, retrospective cohort study of CD patients treated at a single tertiary referral hospital (Amsterdam UMC). All CD patients with an ileostomy were identified by the diagnosis codes for Crohn’s Disease and ileostomy (K50 and Z93.3, resp., International Classification of Diseases, Tenth Edition [ICD-10]) from 1988 until August 2023. Clinical data were collected at CD diagnosis, at the time of ileostomy placement and during postoperative follow-up. Patients were included if they met the following inclusion criteria: an established diagnosis of CD and a long-term ileostomy, which was defined as the presence of an ileostomy for at least 12 months. Patients aged ≤16 years at time of surgery and patients with a diagnosis of ulcerative colitis were excluded. Manual chart review was performed for all eligible patients. Patients who participated in the Institutional Review Board–approved Future IBD biobank (NL53989.018.15) already gave consent for data usage for research purposes. The remaining patients received a consent letter with the option to opt-out.

### Outcomes

Our primary outcome was the occurrence of ileitis proximal to the ileostomy, defined as the presence of aphthous lesions and/or ulcerations at endoscopy (ie, ulcer subscore ≥1 in the Simple Endoscopic Score for Crohn’s Disease, SES-CD). All ileoscopy procedures were performed with a flexible endoscope (gastroscope or pediatric colonoscope) and had documentation with photos of normal and inflamed ileal mucosa. Bowel preparation was as per routine clinical practice and consisted of a low-fiber diet and oral PEG.

Additional outcomes included to evaluate the accuracy of fecal calprotectin in detecting endoscopic disease activity and to assess the endoscopic remission rates after treatment (ie, SES-CD ulcer subscore of 0).

### Variables

Patient demographics and disease characteristics at diagnosis and at time of ileostomy placement were collected from the medical records, including CD phenotype at diagnosis (Montreal classification^12^), surgical history, prior medical treatment, smoking status at time of CD diagnosis and surgery, the indication for ileostomy placement, the extent of intestinal resection, and endoscopic and therapeutic follow-up. Fecal calprotectin levels obtained within a 4-week window before or after ileoscopy were collected.

To account for heterogeneity in treatment pathways, a subgroup of patients with ileostomy placement after the year 2000 was created. This was chosen to delineate the availability of infliximab in routine clinical practice so that all included patients could have been exposed to biologics.

Risk factors for postoperative disease recurrence were based on the literature for postoperative management in CD patients with ileocolonic anastomosis. The following factors were included age at diagnosis ≤16 years, active smoking, penetrating phenotype, history of IBD-surgery, and inflammation at resection margin.^13^ Presence of ileostomy outlet stenosis was evaluated by digital palpation before endoscopy.

### Statistical Analysis

Descriptive analysis was used to examine the patient demographics and disease characteristics. Continuous variables were expressed as mean with standard deviation or as median with interquartile range (IQR), depending on the distribution of the variables. Categorical variables were expressed as proportions. Kaplan–Meier curves were constructed to assess postoperative recurrence and compared using the log-rank test. Patients with missing endoscopy data were excluded from this analysis. Cox regression analysis with time from surgery to endoscopic recurrence was performed to identify independent predictors of recurrent disease; results were expressed as a hazard ratio (HR) with 95% confidence interval (CI). Variables with *P* < .10 were used for multivariate analysis. *P* ≤ .05 was considered to be significant. Receiver-operating characteristic analysis was used to evaluate diagnostic accuracy of fecal calprotectin. Statistical analysis was performed in SPSS Statistics version 29.0 (IBM Corp).

## Results

We identified 150 patients meeting the inclusion criteria, of whom 96 were women (64.0%). The baseline demographic and clinical characteristics are presented in Table 1. The median age at diagnosis was 22 years, and the median disease duration at ileostomy placement was 7 years. Most patients had colonic involvement (82.0%), which was predominantly isolated colonic (L2) CD. Almost half of the cohort had perianal involvement. Two-thirds of the patients had already been treated with biologics prior to surgery, with prior exposure to an anti-TNF agent in all but 1 patient. A quarter of patients were active smokers at the time of ileostomy placement. Ileostomy placement occurred after the year 2000 in 117 patients (78.0%). Main indications were refractory disease (55.3%), perforation (28.0%), and/or strictures (7.3%). At the time of ileostomy placement, 70 patients had already undergone a resection of ≥1 colonic segment. An additional 45 patients had (part of) their colon removed during ileostomy surgery (proctocolectomy *n* = 27, subtotal colectomy *n* = 48, segmental colectomy *n* = 18, ileocolonic resection *n* = 22). A quarter of patients were on biologic therapy during surgery (*n* = 46), of whom 27 continued post ileostomy placement (58.7%).

**Table 1.** Demographic and clinical characteristics (*n* = 150).

Female, *n* (%)	96 (64.0)
Median age at diagnosis (years)	22.0 (16.0-31.0)
Median disease duration at time of surgery (years)	7.0 (2.0-15.0)
Median age at time of surgery (years)	34.0 (24.0-47.3)
Smoking status at time of surgery, *n* (%)	
Never	80 (53.3)
Former	27 (18.0)
Current	35 (23.3)
Unknown	8 (5.3)
Disease location at diagnosis^a^, *n* (%)	
L1, ileal	13 (8.7)
L2, colonic	82 (54.7)
L3, ileocolonic	41 (27.3)
Unknown	14 (9.3)
Disease behavior at diagnosis^a^, *n* (%)	
B1, inflammatory	78 (52.0)
B2, stricturing	11 (7.3)
B3, penetrating	56 (37.3)
Unknown	5 (3.3)
Perianal disease at time of surgery, *n* (%)	64 (42.7)
Previous intestinal resection, *n* (%)	78 (52.0)
Previous immunomodulator, *n* (%)	102 (68.0)
Previous biological therapy, *n* (%)	96 (64.0)
Infliximab or adalimumab	95 (63.3)
Vedolizumab	20 (13.3)
Ustekinumab	18 (12.0)
≥2 prior biologicals	29 (19.3)
Ileostomy placement after 2000, *n* (%)	117 (78.0)
≥1 conventional risk factor^b^, *n* (%)	125 (83.3)

^a^According to the Montreal Classification.

^b^Defined as age at diagnosis ≤16 years, active smoking, penetrating phenotype, history of IBD-surgery and inflammation at resection margin.

### Endoscopy

**Figure 1.**
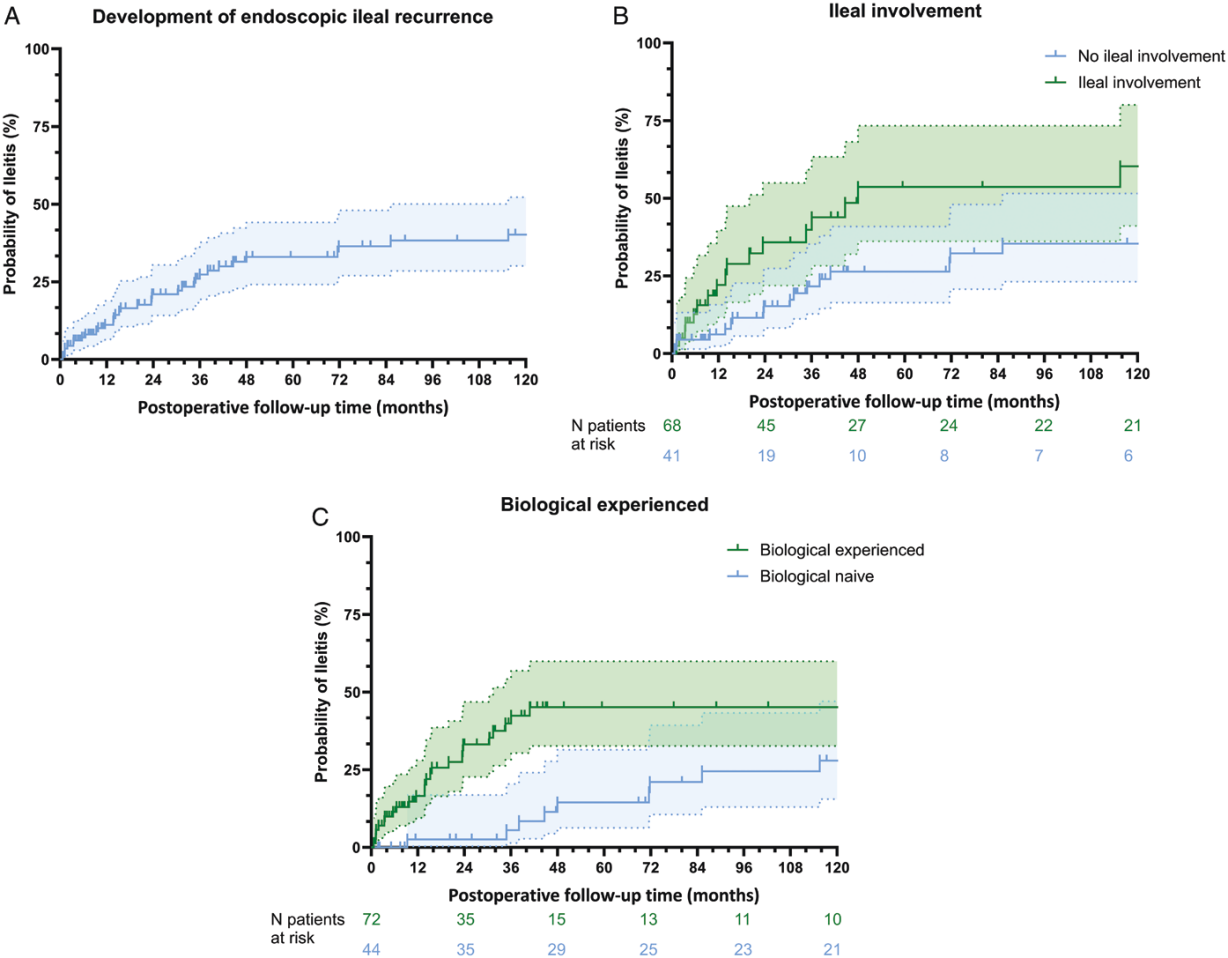
(A) Kaplan–Meier analysis of the development of endoscopic ileal recurrence (overall). (B) Kaplan–Meier analysis of the development of endoscopic ileal recurrence in patients with prior ileal involvement. Log-rank test *P* = .016. (C) Kaplan–Meier analysis of the development of endoscopic ileal recurrence in bio-experienced patients. Log-rank test *P* = .003.

Of the 150 patients, 116 (77.3%) underwent endoscopic assessment during a median follow-up of 6.0 years (IQR 2.0-14.0). Patient characteristics are summarized in Table S1. In 73 of the 116 patients, 2 or more endoscopies were performed postoperatively, with a median time of 34.7 months (IQR 10.0-132.1) between surgery and the first endoscopy. Indications for ileoscopy were clinical symptoms (50.9%), routine follow-up (20.7%), and evaluation before potential ostomy reversal (14.7%). Endoscopic recurrence (ie, presence of aphthous or ulcerative lesions) was detected in 46/116 patients during follow-up, of which 28 (24.1%) at the first ileoscopy after ileostomy placement. Large ulcers (≥0.5 cm diameter) were seen in 27/46 patients. Ten patients presented with an ileostomy outlet stenosis at their first endoscopy, but this was not associated with a higher ulcer recurrence rate (*P* = .24). Figure 1 shows the time from surgery to endoscopic disease recurrence in patients who underwent endoscopic assessment. The overall recurrence rate was 11.2%, 27.3%, and 33.0% after 1, 3, and 5 years, respectively, with a median time to detection of ileitis of 147.7 months. Patients with ileal involvement and patients with prior exposure to biologics had higher recurrence rates at 22.1%, 43.9%, and 53.7% (log rank *P* = .016), and 16.6%, 42.2%, and 45.1% (log rank *P* = .003), respectively. Bio-experienced patients with ileal involvement had the highest recurrence rate at 27.5% at 1 year and 64.2% at 3 years, with a median time to detection of ileitis of 34.6 months (log rank *P* < .001).

Ileal involvement, preoperative exposure to biologics, and male gender were associated with the detection of ileitis on univariate regression analysis. On multivariate analysis, ileal involvement (HR 1.99, 95% CI 1.09-3.62; *P* = .02) and prior biological therapy (HR 2.48, 95% CI 1.25-4.89; *P* = .01) could be confirmed as independent predictors of endoscopic disease recurrence (Table 2). In a subgroup analysis evaluating patients with ileostomy placement in the biologic era (after the year 2000, n = 117), only ileal involvement remained an independent risk factor (HR 2.80, 95% CI 1.31-6.00; *P* = .008) (Table S2). The proportion of patients developing ileitis was similar between those who underwent proctocolectomy with end ileostomy and those who did not (*P* = .40). This lack of association was further supported by Cox regression analysis (HR 0.74, 95% CI 0.34-1.60; *P* = .44).

**Table 2.** Risk factors for endoscopic disease recurrence.

	Univariate analysis		Multivariate analysis	
Variable	Hazard ratio (95% CI)	*P*	Hazard ratio (95% CI)	*P*
≤16 years at diagnosis	0.76 (0.39-1.50)	.42	—	
Male gender	1.70 (0.94-3.08)	.08	1.57 (0.86-2.85)	.15
Smoking status at the time of surgery			—	
Never	*Ref.*			
Former	1.21 (0.46-3.19)	.58		
Current	1.21 (0.62-2.37)	.70		
Disease behavior^a^			—	
Inflammatory	*Ref.*			
Stricturing	0.26 (0.04-1.93)	.19		
Penetrating	0.81 (0.35-1.90)	.63		
Ileal involvement^a^	2.05 (1.13-3.72)	.018	1.99 (1.09-3.62)	.024
Perianal disease at time of surgery	1.18 (0.64-2.20)	.59	—	
Prior biological therapy	2.64 (1.35-5.18)	.005	2.48 (1.25-4.89)	.009
Prior intestinal resection	1.50 (0.84-2.70)	.17	—	
Presence of ≥1 conventional risk factor^b^	0.85 (0.42-1.73)	.66	—	
Ileostomy placement after 2000	1.74 (0.88-3.46)	.11	—	
Prophylactic immunomodulator/biologic	1.01 (0.50-2.03)	.98	—	
Disease activity in resection margins	0.52 (0.18-1.52)	.24	—	

Abbreviation: CI, confidence interval.

^a^According to the Montreal Classification.

^b^Defined as age at diagnosis ≤16 years, active smoking, penetrating phenotype, history of IBD-surgery and inflammation at resection margin.

### Fecal Calprotectin

Fecal calprotectin levels obtained within 4 weeks of endoscopy were available for 37 patients. Patients in endoscopic remission (*n* = 19) had significantly lower median calprotectin levels (21 µg/g, IQR 10-94) compared to those with active disease (*n* = 18; 418 µg/g, IQR 165-1076; *P* < .001). Patients with large to very large ulcers (≥0.5 cm) (*n* = 13; 720 µg/g, IQR 327-1331) had significantly higher median calprotectin levels than patients with only aphthous lesions (*n* = 5; 184 µg/g, IQR 63-267; *P* = .034, Figure S1).

**Figure 2.**
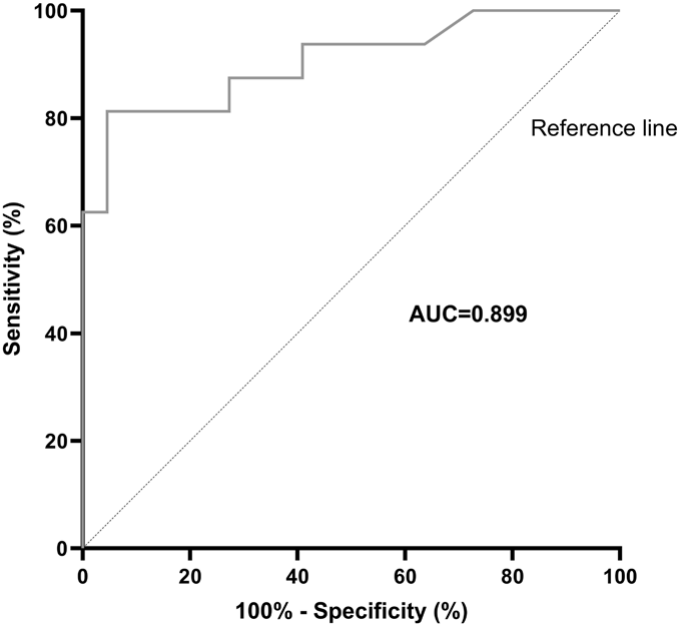
Diagnostic performance of fecal calprotectin in assessing endoscopic disease activity: Receiver-operating characteristic curve analysis. AUC, area under the curve.

At a cutoff of 170 µg/g, fecal calprotectin demonstrated good diagnostic accuracy for detecting endoscopic inflammation, with a sensitivity of 77.8%, a specificity of 94.7%, yielding a positive predictive value (PPV) of 90.6% and a negative predictive value (NPV) of 86.6% (Figure 2 and Table S3). A lower cutoff of 95 µg/g effectively excluded ileitis, achieving a sensitivity of 88.9% and a specificity of 78.9% (NPV 91.5%, PPV 73.5%) (AUC 0.90, 95% CI 0.80-1.00; *P* < .001).

### Surgery

In total, 101 patients (67.3%) were reoperated, and bowel continuity was ultimately restored in 29 patients (19.3%), of whom 5 needed ileostomy reconstruction first. Forty-two patients underwent ≥2 reoperations. The median time to reconstruction was 29.8 months (IQR 9.0-74.2) and reversal 31.3 months (IQR 17.5-59.9).

Indications for the first ileostomy reoperation were active CD in 26 (25.7%) patients (8 colonic disease activity, 18 ileal disease activity), mechanical complications in 45 (44.6%) patients, ileostomy reversal in 24 (23.8%) patients, miscellaneous in 2 patients (trauma, pyoderma gangrenosum), and unspecified in 4 patients.

Thirty of the 33 patients with a derivative ileostomy were reoperated. Ten patients (30.3%) had their ileostomy reversed, and 23 underwent reconstructive surgery. Ileostomy reconstruction was performed for mechanical complications in 6 patients, for ileal CD recurrence in 5, for unspecified reasons in 2, and other indications in 2 (trauma, pyoderma gangrenosum). Six patients (18.2%) needed a proctocolectomy for refractory colonic CD.

### Medical Treatment

In 42 patients (28.0%), immunomodulators and/or biologics were continued postoperatively. Biologics were continued after ileostomy placement in 27/46 patients on biologics preoperatively. Prophylactic treatment was not associated with a lower the risk of disease recurrence (HR 1.01 [0.50-2.03]; *P* = .98).

In 33 patients with endoscopic ileitis (71.7%), biologic treatment was initiated. Among them, 16 experienced treatment failure with at least 1 agent. Specifically, 14 patients received infliximab, 15 patients received adalimumab, 11 patients received vedolizumab, and 13 received ustekinumab. Median treatment duration was 35.0 months (IQR 16.5-46.0), 9.0 months (IQR 4.0-60.5), 49.0 months (IQR 13.0-100.5), and 18.5 months (IQR 5.0-60.0), respectively. Half of the patients (13/23) in whom anti-TNF treatment was started for postoperative recurrence had already received at least 1 anti-TNF agent prior to ileostomy placement. Endoscopic remission was achieved in 20/33 patients (60.6%): 5/14 with infliximab, 8/15 with adalimumab, 8/11 with vedolizumab, and 3/13 with ustekinumab (Figure S2).

### Sensitivity Analysis

In our cohort, 34 patients (22.7%) did not undergo endoscopic follow-up after ileostomy placement. Patient characteristics of this subgroup are summarized in Table S4. These patients were significantly older at diagnosis (median 28.0 years [IQR 22.0-40.3] vs. 22.0 [16.0-30.0]; *P* = .010) and at time of surgery (median 43.0 years [31.3-55.5] vs. 32.0 [22.3-43.0]; *P* < .001). Exact disease location was unknown in 7/34 versus 7/116 (*P* = .018). Ileostomy reconstruction and reversal rate were comparable between both groups.

During follow-up, 2/34 patients experienced clinically active disease as assessed by their treating physician. Fecal calprotectin levels were unavailable in the majority of patients. Considering that the endoscopic recurrence rate for symptomatic patients was 33.9%, compared to 7.3% in asymptomatic patients undergoing endoscopy for follow-up, the additional number of patients with endoscopic recurrence would be 3/34 and the estimated recurrence rate for the entire study cohort 49/150 (32.7%).

## Discussion

Our understanding of the natural course of postoperative CD recurrence primarily stems from trials involving patients undergoing ileocecal resection, leading to evidence-based guidelines to monitor and treat CD patients with an ileocolonic anastomosis.^10,11,14^ However, knowledge regarding disease behavior and the efficacy of current therapies in CD patients with a permanent ileostomy remains scarce. This study, representing the largest series to date on endoscopic disease activity in patients with a long-term ileostomy, reports that one-third of patients who underwent endoscopic evaluation develop ileal recurrence within 5 years of surgery.

Prior studies have predominantly focused on clinical and/or surgical endpoints, leaving the endoscopic disease course in CD patients with an ileostomy unknown. A recent meta-analysis encompassing 14 trials and 1004 patients documented a median clinical recurrence rate of 23.5% (range, 7-35) and 40% (range, 11-60) at 5 and 10 years, respectively.^15^ Endoscopic outcomes were reported in 2 recent trials. In a retrospective series with a median follow-up of 1.6 years by Lopez et al., 12 patients exhibited luminal recurrence at rates of 8%, 16%, and 35% at 1, 2, and 5 years, respectively.^7^ Similarly, Hollis et al. identified 193 patients who underwent proctocolectomy with end ileostomy between 2009 and 2019.^6^ Among these, 57 patients (29.5%) underwent ileoscopy during a median follow-up of 1.8 years. The crude endoscopic recurrence rate was 11.9% with a median time to recurrence of 2.6 years. The estimated overall recurrence rate at 5 years was 40.8%. We confirm these findings in a larger patient cohort and demonstrate a considerably lower recurrence rate (11.2%, 27.3%, and 33.0% after 1, 3, and 5 years, resp.) compared to patients with primary ileocolonic anastomosis.^16^

Consistent with prior findings in 51 ileostomy patients, our study demonstrates the high diagnostic accuracy of fecal calprotectin for identifying endoscopic disease activity (AUC 0.90), with higher levels correlating with more severe inflammation.^17^ These data suggest that a fecal calprotectin range of 95-170 µg/g may serve as a useful noninvasive biomarker to guide the need for endoscopic assessment; however, these findings should be validated in a prospective cohort.

Established clinical risk factors for postoperative recurrence following ileal resection with ileocolonic anastomosis, including active smoking, penetrating disease, and prior intestinal surgery, may not generalize to patients with an ileostomy.^6,7,9,13,18^ The most consistent risk factor for disease recurrence in ileostomy patients is ileal involvement, with more conflicting results on young age at CD diagnosis or surgery, complicated CD phenotype, prior small bowel surgery, and exposure to biologics before ileostomy placement.^15^ In line with this previous research, our analysis failed to confirm these traditional clinical factors as independent risk factors for endoscopic ileal recurrence in patients with an ileostomy. Both the presence of ileal disease and prior use of biologic therapy, however, are independent predictors of luminal recurrence in our cohort. Since a significant proportion of patients had their ileostomy placed before infliximab became available in clinical practice, we performed a subgroup analysis of patients operated after the year 2000, showing potentially a lesser impact of previous biologic exposure.

This study explored the effectiveness of current biologics in treating ileal inflammation in patients with an ileostomy. While the sample size necessitates cautious interpretation, several observations are noteworthy. Continuing prophylactic treatment after surgery in unselected patients did not affect endoscopic disease recurrence in our cohort, mirroring earlier findings.^7^ While most patients failed at least one biologic postoperatively, endoscopic remission could be achieved in 60.6% of patients with ileostomy ileitis. It is possible that the endoscopic remission rate is underestimated, given that some patients in clinical and/or biochemical remission did not undergo endoscopic evaluation of mucosal healing.

There are limitations to this study. Due to its retrospective nature, results are mainly hypothesis-generating and multicenter, prospective trials are needed to definitively assess the role of risk factors, endoscopy, and treatment strategies. As official guidance on how to manage patients with an ileostomy is lacking, the timing of endoscopy, if any, as well as treatment decisions were at the discretion of the treating physicians. Notably, 34 patients in our cohort (22.7%) did not undergo endoscopy, and investigations were predominantly symptom-driven (55.3%) in those who did. This selective approach to endoscopy introduces a potential bias, potentially leading to an overestimation of the true endoscopic recurrence rate within our study. Recurrence rates were calculated only in the subset of patients who underwent endoscopic evaluation, and we are unable to make firm conclusions on the risk of endoscopic recurrence in those who did not. To address this, we conducted a sensitivity analysis estimating the overall endoscopic recurrence rate in our cohort at 32.7%. Given that this analysis relied solely on clinical recurrence, lacking supporting calprotectin data, we anticipate this to be a conservative underestimation. In addition, we chose to include all patients with a long-term ileostomy rather than only patients with a proctocolectomy and end ileostomy. Although this may have introduced heterogeneity in our study population, we believe that this cohort represents a clinically relevant subgroup of patients. Indeed, we show that <20% of patients with an ileostomy for >1 year had their bowel continuity restored and found no evidence of differences in ileal recurrence rate in patients with or without a remnant colon in situ.

## Conclusion

In CD patients with endoscopic evaluation, one-third have endoscopic recurrence within 5 years of ileostomy placement. Patients with ileal involvement or prior exposure to biologics are at increased risk of recurrent disease and may benefit from endoscopic monitoring, given the promising role of current biological therapies in achieving endoscopic remission. Due to its excellent discriminatory ability, we suggest measuring fecal calprotectin in ileostomy effluent every 6 months postoperatively to guide the need for and frequency of endoscopic assessments.

## Supplementary Material

izaf153_Supplementary_Tables_S1-S4

izaf153_Supplementary_Figures_S1

izaf153_Supplementary_Figures_S2

## Data Availability

The data underlying this article cannot be shared publicly in order to protect the privacy of individuals who participated in the study. The data will be shared on reasonable request to the corresponding author.
