# Supplementary material for: Endoscopic Recurrence in Crohn’s Disease Patients With Long-Term Ileostomy
Source: Inflamm Bowel Dis. 2025 Jul 12;31(12):3298–304. doi: 10.1093/ibd/izaf153 (PMC12688077; doi:10.1093/ibd/izaf153)
Supplement: izaf153_Supplementary_Tables_S1-S4 [file izaf153_supplementary_tables_s1-s4.docx]

**SUPPLEMENTARY MATERIAL**

**Table S1. Ileostomy placement in the biological era**

| **ST A1.** Demographic and clinical characteristics | |  |
| --- | --- | --- |
|  | All patients  (n = 150) | Ileostomy after 2000  (n = 117) |
| Female, n (%) | 96 (64.0) | 76 (65.0) |
| Median age at diagnosis (y) | 22.0 (16.0-31.0) | 23.0 (17.0-32.0) |
| Median disease duration at time of surgery (y) | 7.0 (2.0-15.0) | 8.5 (2.8-18.0) |
| Median age at time of surgery (y) | 34.0 (24.0-47.3) | 36.5 (26.8-50.0) |
| Smoking status at time of surgery, n (%)  Never  Former  Current  Unknown | 80 (53.3)  27 (18.0)  35 (23.3)  8 (5.3) | 62 (53.0)  26 (22.2)  23 (19.7)  6 (5.1) |
| Disease location at diagnosis *, n (%)  L1, ileal  L2, colonic  L3, ileocolonic  Unknown | 13 (8.7)  82 (54.7)  41 (27.3)  14 (9.3) | 9 (7.7)  64 (54.7)  33 (28.2)  11 (9.4) |
| Disease behaviour at diagnosis *, n (%)  B1, inflammatory  B2, stricturing  B3, penetrating  Unknown | 78 (52.0)  11 (7.3)  56 (37.3)  5 (3.3) | 62 (53.0)  7 (6.0)  44 (37.6)  4 (3.4) |
| Perianal disease at time of surgery, n (%) | 64 (42.7) | 53 (45.3) |
| Previous intestinal resection, n (%) | 78 (52.0) | 60 (51.3) |
| Previous immunomodulator, n (%) | 102 (68.0) | 93 (79.5) |
| Previous biological therapy, n (%)  Infliximab or adalimumab  Vedolizumab  Ustekinumab  ≥ 2 prior biologicals | 96 (64.0)  95 (63.3)  20 (13.3)  18 (12.0)  29 (19.3) | 95 (81.2)  94 (80.3)  20 (17.1)  18 (15.4)  29 (24.8) |
| Ileostomy placement after 2000, n (%) | 117 (78.0) | 117 (100) |
| ≥1 conventional risk factor ^±^, n (%) | 125 (83.3) | 97 (82.9) |
| Endoscopic follow-up, n (%) | 116 (77.3) | 89 (76.1) |
| Endoscopic recurrence, n (%) | 46 (30.7) | 31 (23.8) |
| * According to the Montreal Classification ± Defined as age at diagnosis ≤16 years, active smoking, penetrating phenotype, history of IBD-surgery and inflammation at resection margin | | |

**Table S2. Risk factors for endoscopic disease recurrence in patients with ileostomy in the biological era**

| N=117 | Univariate analysis | | Multivariate analysis | |
| --- | --- | --- | --- | --- |
| Variable | Hazard ratio (95% CI) | *P* | Hazard ratio (95% CI) | *P* |
| ≤ 16 years at diagnosis | 0.59 (0.24-1.45) | 0.25 | — |  |
| Male gender | 1.82 (0.86-3.83) | 0.12 | — |  |
| Smoking status at the time of surgery |  |  | — |  |
| Never | *Ref.* |  |  |  |
| Former | 1.14 (0.49-2.65) | 0.76 |  |  |
| Current | 1.14 (0.42-3.12) | 0.79 |  |  |
| Disease behaviour * |  |  | — |  |
| Inflammatory | *Ref.* |  |  |  |
| Stricturing | / | 0.98 |  |  |
| Penetrating | 0.89 (0.30-2.66) | 0.83 |  |  |
| Stenosing or penetrating disease * | 0.62 (0.21-1.84) | 0.39 | — |  |
| Ileal involvement * | 3.11 (1.46-6.66) | 0.003 | 2.80 (1.31-6.00) | 0.008 |
| Perianal disease at time of surgery | 1.04 (0.49-2.18) | 0.93 | — |  |
| Prior biological therapy | 4.83 (1.14-20.47) | 0.03 | 3.45 (0.81-14.74) | 0.10 |
| Prior intestinal resection | 1.53 (0.74-3.15) | 0.25 | — |  |
| Presence of ≥ 1 conventional risk factor ^±^ | 0.78 (0.34-1.83) | 0.57 | — |  |
| Disease activity in resection margins | 0.57 (0.15-2.18) | 0.41 | — |  |
| CI, confidence interval  * According to the Montreal Classification  ± Defined as age at diagnosis ≤16 years, active smoking, penetrating phenotype, history of IBD-surgery and inflammation at resection margin | | | | |

**Table S3. Diagnostic performance of fecal calprotectin in assessing endoscopic disease activity**

| Calprotectin (mcg/g) | Sensitivity | Specificity | Positive predictive value | Negative predictive value |
| --- | --- | --- | --- | --- |
| 52 | 0,889 | 0,632 | 0,613 | 0,896 |
| 73 | 0,889 | 0,684 | 0,649 | 0,904 |
| 86 | 0,889 | 0,737 | 0,689 | 0,910 |
| 95 | 0,889 | 0,789 | 0,735 | 0,915 |
| 101 | 0,833 | 0,789 | 0,722 | 0,878 |
| 123 | 0,778 | 0,789 | 0,708 | 0,844 |
| 145 | 0,778 | 0,842 | 0,764 | 0,852 |
| 154 | 0,778 | 0,895 | 0,829 | 0,860 |
| 171 | 0,778 | 0,947 | 0,907 | 0,866 |
| 212 | 0,722 | 0,947 | 0,900 | 0,838 |

**Table S4. Demographic and clinical characteristics of patients with or without endoscopic follow-up**

|  | All patients  (n = 150) | Ileostomy after 2000  (n = 117) | P |
| --- | --- | --- | --- |
| Female, n (%) | 77 (66.4) | 19 (55.9) | 0.262 |
| Median age at diagnosis (y) | 22.0 (16.0-30.0) | 28.0 (22.0-40.3) | 0.010 |
| Median disease duration at time of surgery (y) | 7.0 (2.0-13.0) | 10.5 (3.8-18.0) | 0.064 |
| Median age at time of surgery (y) | 32.0 (22.3-43.0) | 43.0 (31.3-55.5) | < 0.001 |
| Smoking status at time of surgery, n (%)  Never  Former  Current  Unknown | 68 (58.6)  26 (22.4)  17 (14.7)  5 (4.3) | 12 (35.3)  10 (29.4)  9 (26.5)  3 (8.8) | 0.070 |
| Disease location at diagnosis *, n (%)  L1, ileal  L2, colonic  L3, ileocolonic  Unknown | 11 (9.5)  68 (58.6)  30 (25.9)  7 (6.0) | 2 (5.9)  14 (41.2)  11 (32.4)  3 (8.8) | 0.671  0.317  0.180  0.018 |
| Disease behaviour at diagnosis *, n (%)  B1, inflammatory  B2, stricturing  B3, penetrating  Unknown | 54 (46.6)  7 (6.0)  20 (17.2)  35 (30.2) | 11 (32.4)  4 (11.8)  4 (11.8)  15 (44.1) | 0.219 |
| Perianal disease at time of surgery, n (%) | 52 (44.8) | 12 (35.3) | 0.334 |
| Previous intestinal resection, n (%) | 56 (48.3) | 22 (64.7) | 0.092 |
| Previous immunomodulator, n (%) | 80 (69.0) | 22 (64.7) | 0.640 |
| Previous biological therapy, n (%)  Infliximab or adalimumab  Vedolizumab  Ustekinumab  ≥ 2 prior biologicals | 72 (62.1)  71 (61.2)  15 (12.9)  13 (11.2)  23 (19.8) | 24 (70.6)  24 (70.6)  5 (14.3)  5 (14.3)  6 (17.6) | 0.363  0.318  0.789  0.581  0.777 |
| Ileostomy placement after 2000, n (%) | 89 (76.7) | 28 (82.4) | 0.486 |
| ≥1 conventional risk factor ^±^, n (%) | 97 (83.6) | 28 (82.4) | 0.862 |
| Endoscopic follow-up, n (%) | 116 (100) | 0 (0) |  |
| Endoscopic recurrence, n (%) | 46 (39.7) | - |  |
| Ileostomy reversal, n (%)  Ileostomy reconstruction, n (%) | 21 (18.1)  63 (54.3) | 8 (23.5)  14 (41.2) | 0.481  0.178 |
| * According to the Montreal Classification ± Defined as age at diagnosis ≤16 years, active smoking, penetrating phenotype, history of IBD-surgery and inflammation at resection margin | | |  |
