# Supplementary figures and images for: Endoscopic Recurrence in Crohn’s Disease Patients With Long-Term Ileostomy
Source: Inflamm Bowel Dis. 2025 Jul 12;31(12):3298–304. doi: 10.1093/ibd/izaf153 (PMC12688077; doi:10.1093/ibd/izaf153)

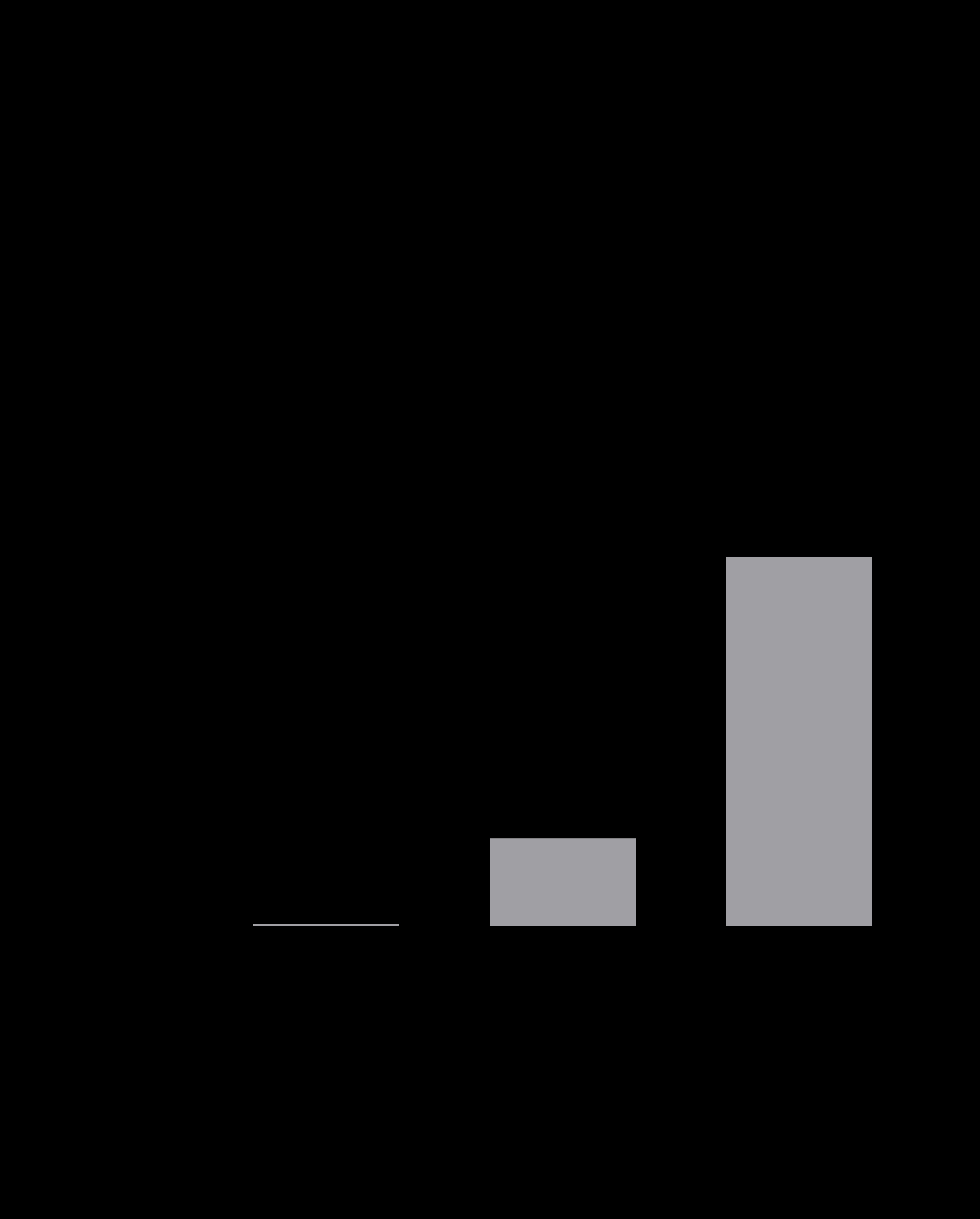

Supplement: izaf153_Supplementary_Figures_S1 [file izaf153_supplementary_figures_s1.jpeg]

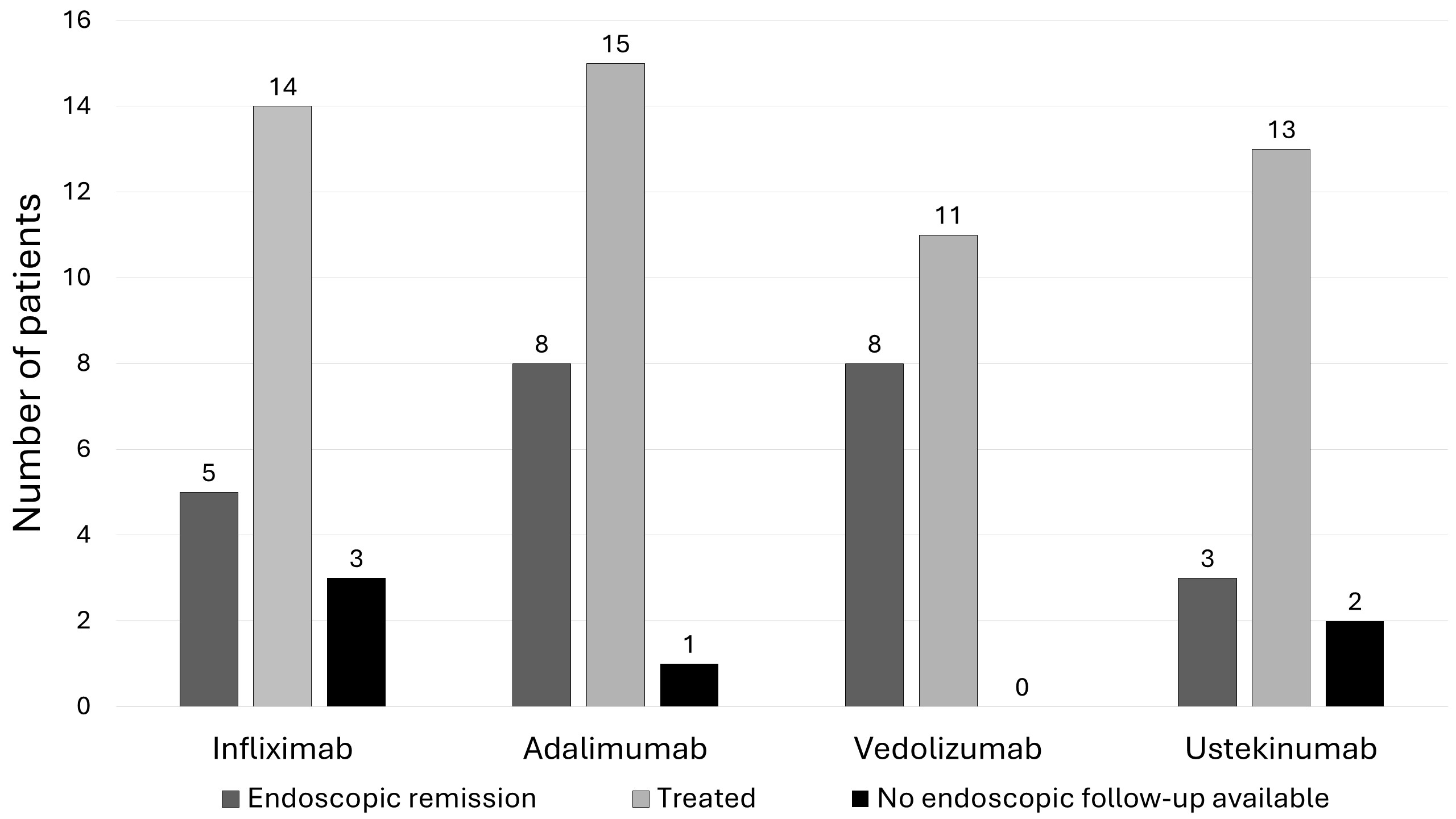

Supplement: izaf153_Supplementary_Figures_S2 [file izaf153_supplementary_figures_s2.jpeg]
